# Research on Portal Venous Hemodynamics and Influencing Factors of Portal Vein System Thrombosis for Wilson’s Disease after Splenectomy

**DOI:** 10.3389/fsurg.2022.834466

**Published:** 2022-05-30

**Authors:** Zhou Zheng, Qingsheng Yu, Hui Peng, Wanzong Zhang, Yi Shen, Hui Feng, Long Huang, Fuhai Zhou, Qi Zhang, Qin Wang

**Affiliations:** ^1^ The First Affiliated Hospital of Anhui University of Chinese Medicine, Hefei, China; ^2^ Institute of Chinese Medicine Surgery, Anhui Academy of Chinese Medicine, Hefei, China

**Keywords:** portal vein system thrombosis (PVST), Wilson’s disease, splenectomy, hypertension, hemodynamic

## Abstract

**Objective:**

Splenectomy is one crucial solution for hypersplenism with portal hypertension. However, portal vein system thrombosis (PVST) caused by hemodynamic changes affects the prognosis of patients. We analyze the changes in portal vein hemodynamics following splenectomy for Wilson’s disease combined with portal hypertension and the influencing factors that lead to PVST.

**Methods:**

A retrospective cohort study was conducted, in which 237 Wilson’s disease patients with hypersplenism underwent splenectomy. The hemodynamic indices of the portal vein were monitored before surgery and on the 1st, 7th, and 14th days around surgery. The patients were divided into PVST and non-PVST groups. The clinical factors were identified by univariate and multivariate logistic regression. The Logit *P* was calculated according to the logistic regression prediction model, and the ROC curve for each independent factor was plotted.

**Results:**

The portal vein velocity, flow, and inner diameter showed a downward trend around surgery, with statistically significant differences between each time point (*P *< 0.01). The PVST incidence rate was 55.7%. Univariate analysis revealed that the platelet (PLT) levels on the postoperative 3rd and 7th days (*P *= 0.001; *P *< 0.001), D-dimer (D-D) on the postoperative 7th and 14th days (*P *= 0.002; *P *< 0.001), preoperative portal vein velocity, flow, diameter (*P *< 0.001), and splenic vein diameter (*P *< 0.001) were all statistically and significantly different between the two groups. Multivariate logistic regression revealed a significant increase in PLT on the postoperative 7th day (OR* *= 1.043, 95% CI, 1.027–1.060, *P *< 0.001) and D-D on the postoperative 14th day (OR = 1.846, 95% CI, 1.400–2.435, *P *< 0.001). Preoperative portal and splenic vein diameters (OR = 1.565, 95% CI, 1.213–2.019, *P *= 0.001; OR = 1.671, 95% CI, 1.305–2.140, *P *< 0.001) were the risk factors for PVST. However, preoperative portal vein velocity and flow (OR = 0.578, 95% CI, 0.409–0.818, *P *= 0.002; OR* *= 0.987, 95% CI, 0.975–0.990, *P *= 0.046) were protective factors for PVST. Logit *P* was calculated using a logistic regression prediction model with a cut-off value of −0.32 and an area under receiver operating characteristic curve of 0.952 with 88.61% accuracy.

**Conclusions:**

Splenectomy relieves portal hypertension by reducing the hemodynamics index. PVST is linked to multiple factors, including preoperative portal vein diameter, velocity, flow, and splenic vein diameter, especially PLT on the postoperative 7th day and D-D on the postoperative 14th day. The predictive model is accurate in predicting PVST.

## Introduction

Wilson’s disease (WD) is a recessive genetic disease caused by mutations in the copper transport gene *ATP7B* on chromosome 13. The disturbance of ATP enzyme synthesis of copper and ceruloplasmin is caused by its mutation during biliary tract excretion, resulting in copper accumulation in the liver, which leads to mitochondrial damage and lipid oxidation in liver cells, accelerating liver cirrhosis progression (1, 2). Liver cirrhosis causes not only portal hypertension but also secondary splenomegaly and hypersplenism, which seriously affect the life of patients. Currently, internal medicine is mostly treated using copper ion complexing agents such as sodium dimercaptopropane sulfonate, penicillamine, and others. However, the occurrence of “bone marrow suppression” and the presence of hypersplenism complicate the continuation of copper repellent therapy. Therefore, splenectomy is suggested to relieve hypersplenism and achieve lifetime copper repellent treatment for WD patients (3).

Splenectomy, as a spleen hypersplenism treatment secondary to WD cirrhosis and gastric fundus esophageal varices, is the current mainstream therapeutic schedule. However, following splenectomy, forming portal vein system thrombosis (PVST) is the most common and serious complication. With the improvement of clinical examination methods such as Doppler ultrasound and spiral computed tomography (CT), as well as a deeper understanding of thrombosis, the harm caused by PVST and its prognostic influence have garnered considerable attention (4).

Yilmaz et al. reported that PVST incidence was 5.0%–8.0% in the natural course of cirrhosis, reaching 20% after splenectomy (5), possibly due to relatively hidden early symptoms of PVST. There may be non-specific manifestations such as fever, abdominal distension, and pain, which can be misdiagnosed. Liver failure, refractory ascites, strangulated intestinal obstruction, and metabolic acidosis can be caused by lesion range development. In particular, peritonitis can be caused by obstructive bowel perforation, leading to the life-threatening multiple organ dysfunction syndrome (MODS) (6, 7).

According to relevant reports, the portal dynamic indicators of patients are changed accordingly during the perioperative period of similar surgery, which may have guiding significance for evaluating patients’ PVST formation and surgery efficacy (8). Currently, there is no global consensus on the related factors and prevention strategies of PVST. Some scholars suggest that PVST formation might correlate with portal vein hemodynamic indicators during surgery. Numerous studies have focused on hemodynamics following splenectomy in hepatitis B liver cirrhosis patients (9, 10). The present work retrospectively analyzes the portal vein hemodynamic characteristics data of 237 patients with WD liver cirrhosis before and after splenectomy to identify the risk factors and early sensitive predictive indicators of postoperative PVST for clinical reference.

## Materials and Methods

### Study Population and Study Design

This study selected 237 patients diagnosed with Wilson and hypersplenism, followed by secondary splenomegaly, who underwent splenectomy in the General Surgery of the First Affiliated Hospital of Anhui University of Traditional Chinese Medicine from January 1, 2010, to December 31, 2019 (Supplementary Data 1, 2). The inclusion criteria were as follows: (1) Patients diagnosed with WD (serum copper blue 1.6 µmol/24 h, liver copper >250 µg/g, and K-F ring positive). (2) The presence of cirrhotic portal hypertension was confirmed by color Doppler ultrasound, CT, or magnetic resonance imaging (MRI). (3) Patients who had moderate or severe hypersplenism and splenomegaly, and white blood cell (WBC) <3 × 10^9^/L, platelets (PLT) <60 × 10^9^/L. (4) Bone marrow hyperplasia was suggested by bone marrow puncture. (5) The score of preoperative liver function was less than 8 points in Child-Pugh with normal coagulation function and complete clinical data. The exclusion criteria were as follows: (1) Splenic artery embolization or transjugular intrahepatic portosystemic shunt (TIP) was performed before. (2) A combination of serious blood system diseases and immune system diseases such as idiopathic thrombotic purpura and Hodgkin’s lymphoma. (3) Liver cirrhosis caused by hepatitis B virus, alcohol, schistosomiasis, and autoimmune hepatitis. (4) Patients with preoperative PVST and severe extraspinal symptoms that cannot be operated on. The diagnostic criteria were as follows: (1) Diagnostic criteria of WD (11). (2) Diagnostic criteria of portal vein thrombosis (12).

All patients underwent splenectomy by the same group of chief physicians with senior professional titles. Specialized statistical analysts collected data from the hospital’s case database and classified patients into PVST and non-PVST groups based on their postoperative conditions.

### Treatment

During perioperative treatment, Patients received symptomatic treatments such as liver protection and enzyme reduction before the operation. Vitamin K1 was administered to patients with abnormal blood coagulation 1 week before the operation, and patients with Child-Pugh C should be adjusted to Child-Pugh B or A before the operation. Prothrombin complex and antibiotics should be taken prophylactically during operation if it lasts longer than 3 h. All patients underwent splenectomy or splenectomy combined with peripheral vascular devascularization. The enlarged spleen was compressed and dragged out when the exposed splenic artery was ligated, and split-cutting of the upper and lower poles of the spleen vessels was completed following laparotomy separation of the gastrocolonic and gastrosplenic ligaments. If pericardial devascularization is performed, the high esophageal branch, collateral paraesophageal branch, inferior phrenic branch, and peripheral blood vessels within the range of 6–8 cm in the lower esophagus should be dissected. Finally, wound hemostasis and serosal suture were performed. Drainage tubes were placed, and the abdomen was closed layer by layer.

### Outcomes and Assessments

The ACUSON Antares 5.0 MHz widescreen convex array probe was used to dynamically monitor the hemodynamic characteristics of the portal vein before and after operation, including the portal vein diameter, maximum velocity, maximum flow, and the formation range of postoperative PVST. Simultaneously, clinical data on patients were collected, including sex, age, Child-Pugh grade, mode of operation, anatomical splenectomy, body mass index, diabetes mellitus, hypertension, and ascites. In addition, the levels of albumin, aspartate aminotransferase, alanine aminotransferase, and total bilirubin, which indicate the liver function, were detected before the operation. The routine blood tests performed before surgery include WBC and hemoglobin. Partially activated prothrombin time, prothrombin time, international standardized ratio, and fibrinogen were recorded to assess coagulation function before the operation. Perioperative platelet count (PLT), D-D and operation time, and intraoperative blood loss during the operation were documented.

### Statistical Analysis

The data were analyzed using SPSS26.0 (SPSS Inc, Chicago, IL, USA) statistical software, and GraphPad Prism 5.0 was used to draw statistical plots. The normal distribution of measurement data was represented by $\bar{x} \pm s$, and non-normal distribution was expressed by median (interquartile range). Repetitive measurement deviation analysis was employed when multiple paired data sphericity >0.05; otherwise, greenhouse correction was used. In univariate analysis, measurement data were analyzed using the independent *t-*test or Mann–Whitney *U* rank-sum test. Enumeration data were represented by rate (%) and tested by using χ^2^ or Fisher’s exact test. Simple linear regression was adopted for single-factor analysis with measurement data as the dependent variable. The factors of the clinical data were gradually incorporated into a multifactor binomial logistic regression model. According to multivariate analysis results, a logistic regression prediction model was established, a receiver operating characteristic (ROC) curve was drawn, and the area under curve including 95% confidence interval was calculated. Simultaneously, the best demarcation point corresponding to the maximum Youden index (sensitivity + specificity − 1) was taken as the boundary value (cut-off value). All tests were bilateral, and *P *< 0.05 was considered statistically significant.

## Results

### Patient Characteristics

**Figure 1:**
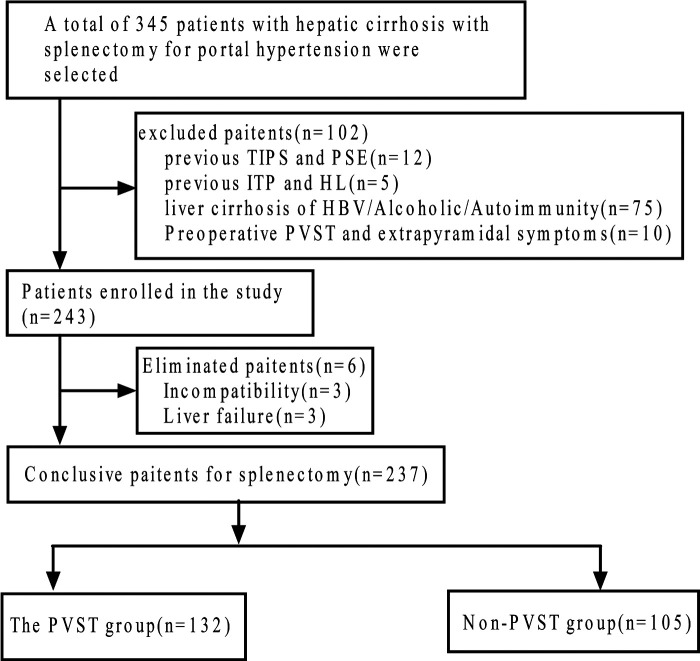
Flow diagram of the study population. TIPS, transjugular intrahepatic portosystemic shunt; PSE, partial splenic embolization; ITP, idiopathic thrombocytopenic purpura; HL, Hodgkin lymphoma; HBV, hepatitis B virus; PVST, portal vein system thrombosis.

PVST occurrence was determined by color Doppler ultrasound or abdominal CT before and 2 weeks after the operation. We screened 237 patients for eligibility and divided them into PVST (*n* = 132) and non-PVST groups (*n* = 105) according to PVST occurrence after the operation, as displayed in Figure 1. Furthermore, patients of the PVST group (67 males, 65 females; age: median age of 29 years ranging from 11 to 63 years; Child-Pugh classes: A 68 and B 64 cases, respectively) and non-PVST group (56 males, 49 females; age: median age of 26 years ranging from 10 to 61 years; Child-Pugh classes: A 68 and B 64 cases, respectively) were included in the research, eventually.

### Hemodynamic Changes in the Portal Vein

A gradual change to a downward trend was observed obviously for patients with regard to portal venous blood velocity, flow, and diameter from the day before surgery to the 3rd, 7th, and 14th days after surgery. A significant alteration in portal vein hemodynamics within pairwise comparison differences between each follow-visiting time point is measured by the effect of time alone in repeated measures (velocity: *F *= 124.31, *P^F ^*< 0.001; flow: *F *= 45.93, *P^F ^*< 0.001; diameter: *F *= 167.66, *P^F ^*< 0.001). Velocity, flow, and diameter maximum values were 22.67 ± 1.46 cm/s, 907.32 ± 37.25 mL/min, and 14.97 ± 2.05 mm, respectively, on the day before surgery, with minimum values of 21.11 ± 1.22 cm/s, 886.28 ± 16.64 mL/min, and 12.83 ± 1.37 mm on the 14th day following surgery (Table 1).

**Table 1:** Postoperative hemodynamic changes in the portal vein.^a^

Hemodynamic	Day (d)				Value						*F*	*P^F^*
	Before	POD3	POD7	POD14	*P*_1 vs.. 3_	*P*_1 vs. 7_	*P*_1 vs. 14_	*P*_3 vs. 7_	*P*_3 vs. 14_	*P*_7 vs. 14_		
Velocity (cm/s)	22.67 ± 1.46	21.46 ± 1.43	21.30 ± 1.32	21.11 ± 1.22	<0.001	<0.001	<0.001	0.023	<0.001	0.006	124.31	<0.001
Flow (ml/min)	907.32 ± 37.25	894.92 ± 38.56	890.52 ± 26.83	886.28 ± 16.64	<0.001	<0.001	<0.001	0.001	<0.001	0.003	45.93	<0.001
Diameter (mm)	14.97 ± 2.05	13.15 ± 2.30	12.99 ± 1.72	12.83 ± 1.37	<0.001	<0.001	<0.001	0.045	0.002	0.007	167.66	<0.001

^*a*^ *Data are indicated as mean ± standard deviation. F and P^F^ denote the difference of different levels when the effect of time alone in vivo was repeatedly measured.*

*Before, preoperative day; POD, postoperative day;*

### Thrombosis of the Portal Vein System

**Figure 2:**
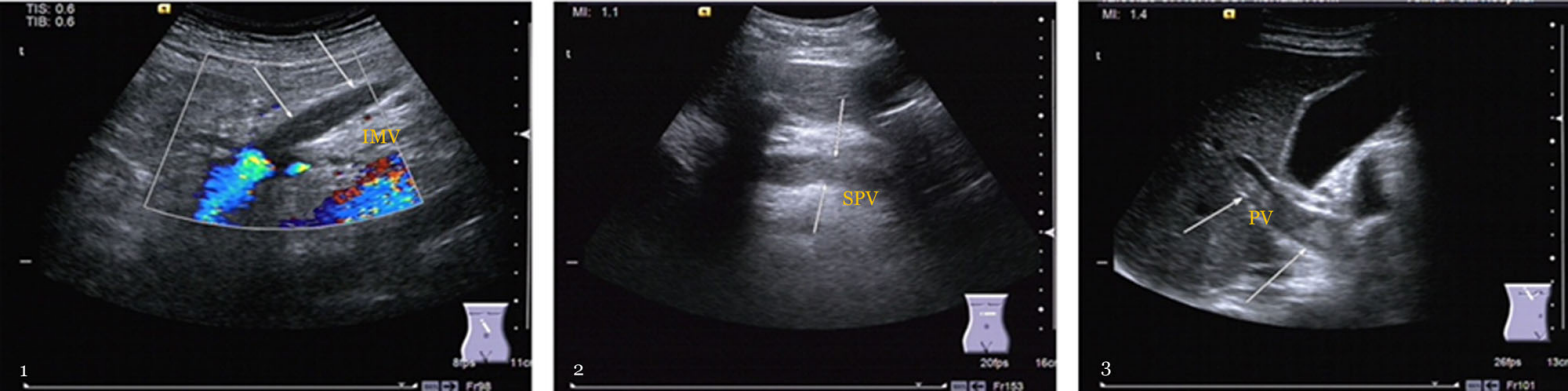
The formation incident of portal vein system thrombosis. 1. Mesenteric vein thrombosis (MVT): gray-scale b-ultrasound image reveals hypoechoic thrombosis within the lumen, and color Doppler ultrasound shows a filling defect in the inferior mesenteric vein (IMV) (arrows). 2. Splenic vein thrombosis: the gray-scale b-ultrasound image shows that splenic vein (SPV) behind the pancreas were filled with hypoechoic thrombus (arrows), and the lumen was blocked. 3. Portal vein thrombosis (PVT): the gray-scale b-ultrasound image demonstrates that the primary portal vein (PV) is closely full of hypoechoic thrombus (arrows), and the main portal vein is almost completely occluded.

More attention was paid to the postoperative incidence of PVST in 132 of 237 (55.7%) patients from our observation and the distribution of thrombus, as depicted in Table 2 and Figure 2. A particularly commiserative and unexpected finding was that one participant was discharged after secondary suture with the incision dehiscence 14 days after the operation, and three patients died due to pancreatic fistula, outburst infection, and early liver failure.

**Table 2:** The incident of postoperative PVST formation.

The location of the thrombosis	The number of cases	Incidence of thrombosis (%)
PVT	20	8.44
SVT	34	14.35
MVT	15	6.23
PVT, SVT	49	20.68
PVT, MVT	6	2.53
PVT, SVT, MVT	8	3.38

*PVT, portal venous thrombosis; SVT, splenic vein thrombosis; MVT, mesenteric venous thrombosis.*

### Univariate Analysis of Two Groups after Splenectomy

Univariate analysis revealed that the influencing factors of PVST formation included a plasma count of PLT on the 3rd and 7th days after surgery (*t *= 3.369, *P *= 0.001; *t *= −10.567,*P *< 0.001), a plasma level of D-D on the 7th and 14th days after surgery (*t *= −3.137, *P *= 0.002; *t *= 8.276, *P *< 0.001), and portal vein velocity, flow, diameter, and splenic vein diameter before splenectomy (*t *= −6.878, *P *< 0.001; *t *= 4.856; *P *< 0.001; *t *= −5.189, *P *< 0.001; *t *= 7.173, *P *< 0.001). Table 3 illustrates the statistically significant difference between the two groups.

**Table 3:** Univariate analysis of perioperative clinical data of patients in the PVST group and non-PVST group.^a^

Items	PVST group (*n* = 132)	Non-PVST group (*n* = 105)	*t/z/χ* ^2^	*P*
Gender [F/M, *n* (%)]	67(50.8)/65(49.2)	56(53.3)/49(46.7)	0.155	0.693
Age (year)	29.00 (22.00 37.00)	26.00(20.00 32.00)	−1.684	0.092
Child-Pugh [A/B, *n* (%)]	68(51.5)/64(48.5)	66(62.9)/39(37.1)	3.062	0.080
Method of operation [*n* (%)]			0.001	0.974
Splenectomy	118(89.4)	94(89.5)		
SPD	14(10.6)	11(10.5)		
Anatomical splenectomy [yes/no, *n* (%)]	56(42.4)/76(57.6)	49(46.7)/56(53.3)	0.427	0.514
BMI (kg/m^2^)	23.51 ± 2.56	23.54 ± 1.87	−0.107	0.915
Diabetes [yes/no, *n* (%)]	28(21.2)/104(78.8)	19(18.1)/86(81.9)	0.357	0.550
Hypertension [yes/no, *n* (%)]	24(18.2)/108(81.8)	16(15.2)/89(84.8)	0.361	0.548
Ascites [yes/no, *n* (%)]	49(37.1)/83(62.9)	33(31.4)/72(68.6)	0.838	0.360
ALB (g/L)	37.74 ± 4.49	37.95 ± 4.83	−0.355	0.723
AST (U/L)	34.92 ± 8.28	34.38 ± 5.03	0.623	0.534
ALT (U/L)	28.00(20.00 37.00)	28.00(19.50 38.00)	−0.180	0.857
WBC (×10^9^/L)	2.67 ± 0.87	2.63 ± 0.81	0.357	0.721
HB (g/L)	107.23 ± 11.55	107.73 ± 8.17	−0.389	0.698
FIB (g/L)	1.66 ± 0.26	1.69 ± 0.18	−1.038	0.300
TBIL (µmmol/L)	27.72 ± 5.00	27.12 ± 4.9	0.926	0.356
APTT (s)	43.25(36.80 48.95)	40.60(36.95 46.00)	−1.103	0.270
PT (s)	14.92 ± 1.96	14.64 ± 1.42	1.285	0.200
INR	1.35 ± 0.15	1.34 ± 0.14	0.529	0.597
PLT (×10^9^/L)				
Before	58.16 ± 7.02	57.39 ± 9.16	0.709	0.479
POD1	140.89 ± 10.84	141.90 ± 31.60	−0.310	0.757
POD3	224.95 ± 17.27	218.64 ± 11.45	3.369	0.001
POD7	487.17 ± 35.40	441.07 ± 30.61	−10.567	<0.001
POD14	567.64 ± 24.58	559.87 ± 41.30	1.704	0.090
POD21	446.55 ± 44.04	446.85 ± 65.00	−0.040	0.968
D-D (mg/L)				
Before	0.66 ± 0.15	0.64 ± 0.17	0.990	0.323
POD1	5.63 ± 0.39	5.59 ± 1.39	0.273	0.785
POD3	9.36 ± 1.21	9.35 ± 1.63	0.079	0.937
POD7	14.52 ± 1.58	13.98 ± 1.02	−3.137	0.002
POD14	17.21 ± 1.67	15.24 ± 1.99	8.276	<0.001
POD21	12.46 ± 2.01	12.32 ± 1.64	0.589	0.556
The time of operation (min)	213.09 ± 6.92	214.57 ± 7.60	−1.565	0.119
Intraoperative bleed loss (mL)	221.01 ± 8.37	220.00 ± 8.15	0.932	0.352
Portal vein diameter (mm)	15.73 ± 1.64	14.01 ± 2.11	−6.878	<0.001
Portal vein velocity (cm/s)	22.27 ± 1.55	23.18 ± 1.16	4.856	<0.001
portal vein flow (ml/min)	897.73 ± 41.46	919.38 ± 26.83	−5.189	<0.001
Splenic vein diameter (mm)	10.89 ± 2.02	9.01 ± 1.95	7.173	<0.001

^*a*^ *Data are indicated as mean ± standard deviation, median (interquartile range) values, or the ratio of patients.*

*PVST, portal vein system thrombosis; SPD, splenectomy with portal devascularization; Before, preoperative day; POD, postoperative day; BMI, body mass index; ALB, albumin; AST, aspartate transaminase; ALT, alanine transaminase; TBIL, total bilirubin; WBC, white blood cell; HB, hemoglobin; APTT, activated partial thromboplastin time; PT, prothrombin time; INR, international standardized ratio; FIB, fibrinogen; PLT, perioperative platelet count; D-D, D-dimer.*

### Perioperative Changes in Platelet and D-D in the Two Groups after Splenectomy

**Figure 3:**
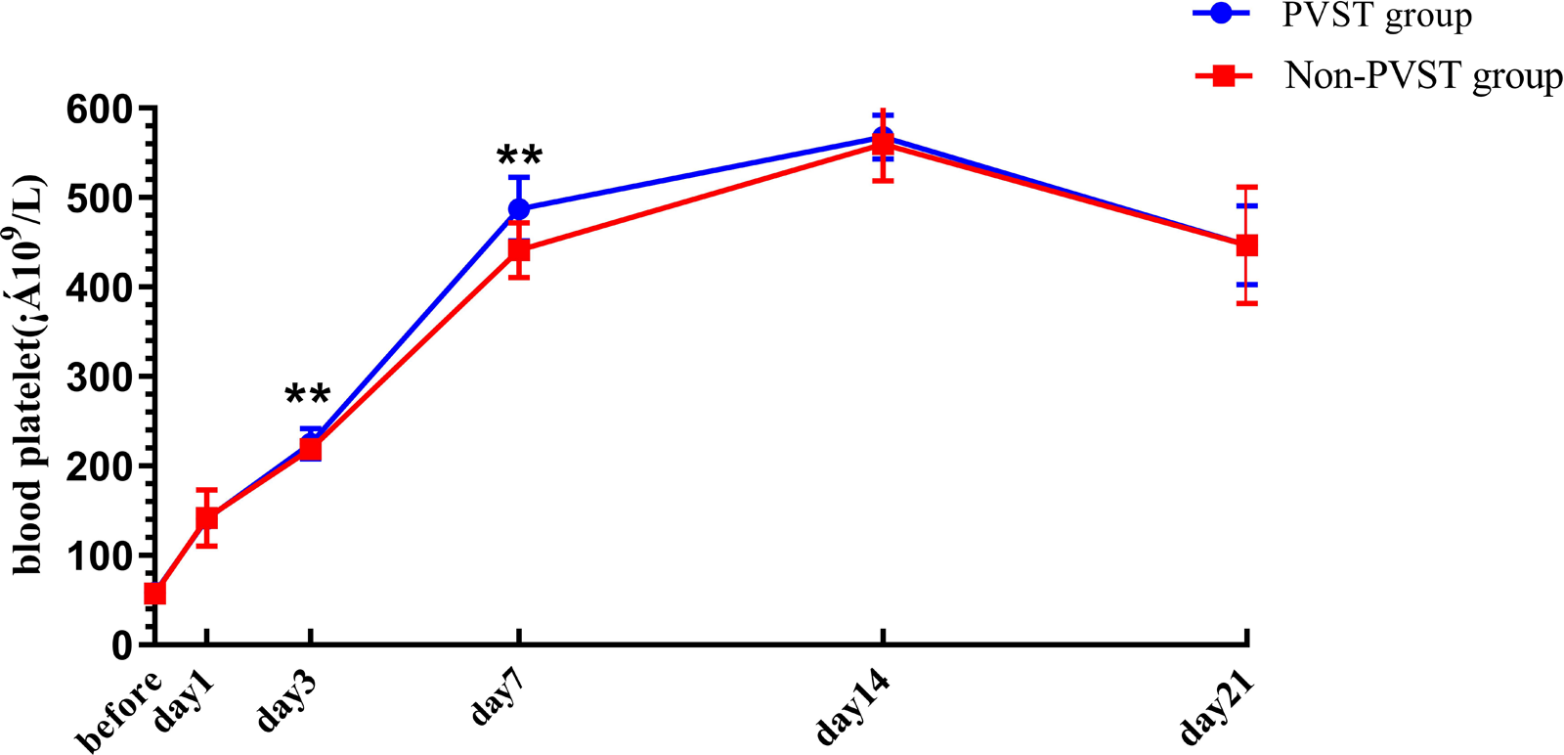
The platelet changes in Wilson’s disease during the perioperative period after splenectomy in the PVST and non-PVST groups. The perioperative changes in plasma platelets in the PVST and non-PVST groups were observed at the 3rd and 7th days after surgery. Data were shown as mean ± SD. *F*-all and *P*-all present the effect of interaction time and group from a repeated measurement linear model time points with differences between the two groups based on model-based pairwise comparisons as simple effects (Bonferroni). The symbol of “**” indicates that the difference between the two groups is statistically significant (*Pp *< 0.01).

**Figure 4:**
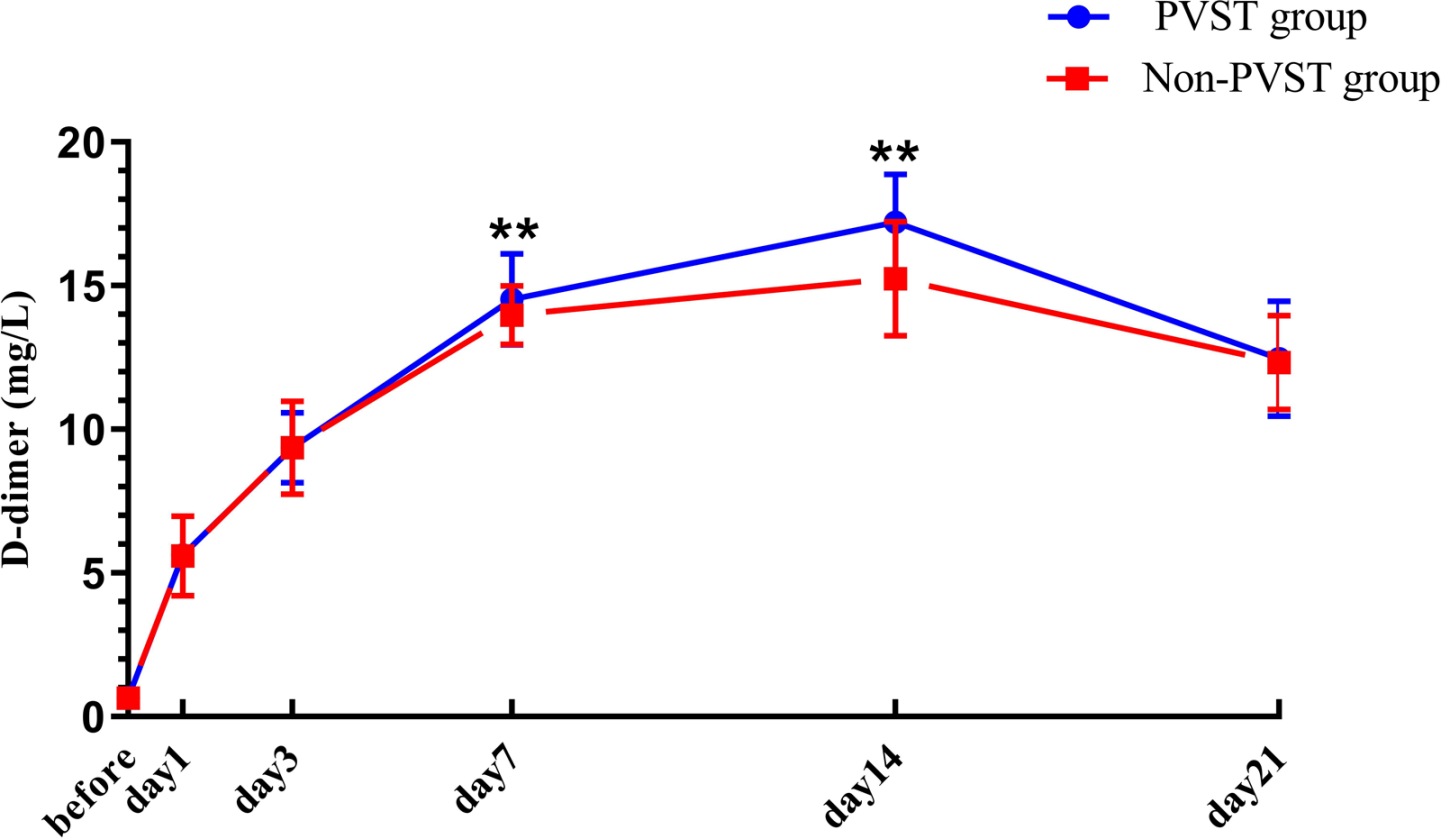
The changes in D-D in Wilson’s disease during the perioperative period after splenectomy in the PVST and non-PVST groups. The perioperative changes in plasma D-D in the PVST and non-PVST groups were observed on the 7th and 14th days after surgery. Data were shown as mean ± SD. F-all and P-all present the effect of interaction time and group from a repeated measurement linear model time points with differences between the two groups based on model-based pairwise comparisons as simple effects (Bonferroni). The symbol of “**” indicates that the difference between the two groups is statistically significant (*Pp *< 0.01).

The preoperative PLT and D-D levels were lower than normal values, and PLT and D-dimer levels increased from the 1st to the 14th day after the operation, reaching a peak on the 14th day after surgery and then decreasing on the 21st day, but both remained higher than normal values. Compared with that in the non-PVST group, the plasma level of PLT in the PVST group significantly increased on the 3rd and 7th days after the operation [day 3: (224.95 ± 17.27) × 10^9^/L vs. (218.64 ± 11.45) × 10^9^/L, *P *= 0.001; day 7: (487.17 ± 35.40) × 10^9^/L vs. (441.07 ± 30.61 × 10^9^/L), *P *< 0.001], and D-D significantly increased on the 7th and 14th days (day 7: 14.52 ± 1.58 mg/L vs. 13.98 ± 1.02 mg/L, *P *= 0.002; 17.21 ± 1.67 mg/L vs. 15.24 ± 1.99 mg/L, *P *< 0.001, respectively) (Figures 3, 4).

### Binary Logistic Multivariate Regressive Analysis of Influential PVST Univariate Factors

The single factor *P *< 0.05 was included in multivariate regression analysis and the results indicated that the plasma levels of PLT and D-D on the 7th and 14th days after the operation (OR* *= 1.043, 95% CI, 1.027–1.060, *P *< 0.001; OR* *= 1.846, 95% CI, 1.400–2.434, *P *< 0.001) and portal vein diameter, velocity, flow, and splenic vein diameter before splenectomy were significantly correlated with postoperative PVST (OR* *= 1.565, 95% CI, 1.213–2.019, *P = *0.001; OR* *= 0.578, 95% CI, 0.409–0.818, *P *= 0.002; OR* *= 0.987, 95% CI, 0.975–0.990, *P *= 0.046*;* OR* *= 1.671, 95% CI, 1.305–2.140, *P *< 0.001), which significantly predict PVST formation (Table 4).

**Table 4:** Logistic multivariate regression analysis of PVST after splenectomy.

Variables	*β*	Se	Wald	df	*P*	OR	95% CI
POD7 PLT (×10^9^/L)	0.042	0.008	28.248	1	<0.001	1.043	1.027–1.060
POD14 D-D (mg/L)	0.613	0.141	18.891	1	<0.001	1.846	1.400–2.435
PVD (mm)	0.448	0.130	11.894	1	0.001	1.565	1.213–2.019
PVV (cm/s)	−0.548	0.177	9.579	1	0.002	0.578	0.409–0.818
PVF (ml/min)	−0.013	0.006	3.987	1	0.046	0.987	0.975–0.990
SVD (mm)	0.513	0.126	16.553	1	<0.001	1.671	1.305–2.140
constant	−16.953	8.426	4.048	1	0.044	0.000	-

*PVST, portal vein system thrombosis; POD, postoperative day; CI, confidence interval; PVD, portal vein diameter; PVV, portal vein velocity; PVF, portal vein flow; SVD, splenic vein diameter.*

### Prediction of Portal Vein System Thrombosis Formation after Operation

**Figure 5:**
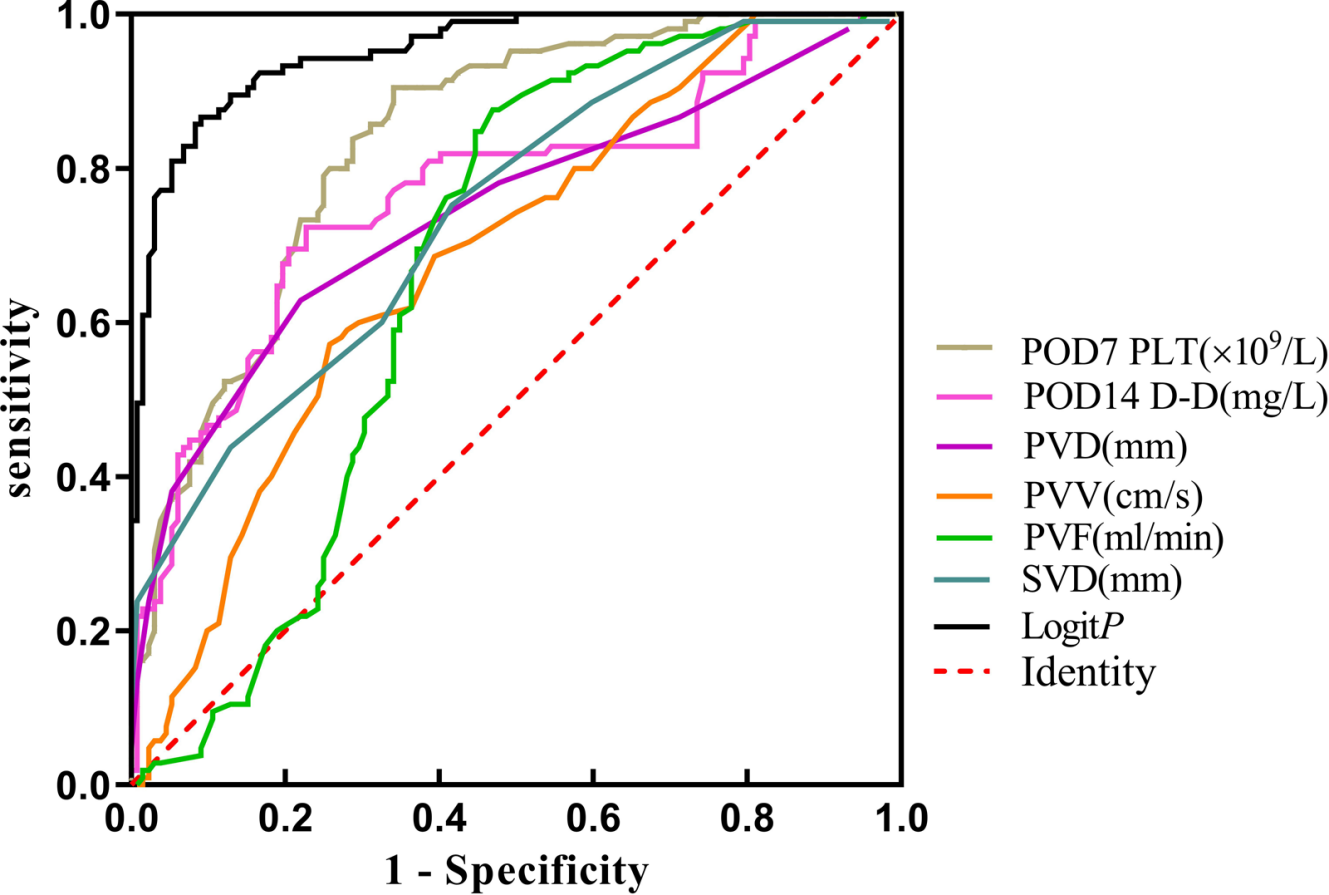
The ROC curve of the PVST predictor after splenectomy for Wilson’s disease cirrhosis with portal hypertension. PVD, portal vein diameter; PVV, portal vein velocity; PVF, portal vein flow; SVD, splenic vein diameter; POD, postoperative day. Logit P: combined prediction of PVST based on meaningful factor.

The Logistic regression prediction model was established from multivariate analysis results: Logit *P* = (−16.953 + 0.042POD7PLT + 0.613POD14D-D + 0.448PVD − 0.548PVV − 0.013PVF + 0.513SVD) (YES = 1, NO = 0). The fact that larger the area under the receiver operating characteristic curve (AUROC) of Logit *P*, the higher the risk of PVST formation after surgery was primarily demonstrated by the ROC curve of Logit *P* and the factors that constitute the model to predict postoperative PVST formation (Figure 5 and Table 5). The AUROC of Logit *P* was 0.952, with the optimal cut-off point corresponding to the maximum Youden index of −0.32, which was significantly higher than the AUROC of each independent risk factor index in the equation. The cut-off points of POD7PLT, POD14D-D, PVD, PVV, PVF, and SVD were 476 × 10^9^/L, 15.99 mg/L, 14 mm, 32.2 cm/sec, 888 mL/min, and 10 mm, respectively. Logit *P *= −0.32 was used as a marker to determine PVST formation after surgery. Among 135 patients, there were 120 with Logit *P *≥ −0.32, and they developed PVST. According to our research, the positive predictive value was 88.89%, and sensitivity was 90.91%. On the other hand, 90 patients out of 102 with Logit *P *< −0.32 did not develop PVST after surgery with a negative predictive value of 88.24% and a specificity of 86.67%. The overall accuracy was 88.61%. Therefore, this kind of situation suggested that Logit *P* has high sensitivity, specificity, and accuracy for postoperative PVST diagnosis (Table 6).

**Table 5:** The area under the receiver operating characteristic curve of PVST.

Variables	AUC	95% CI	*Pp*	Cut-off	Sensitivity (%)	Specificity (%)
POD7 PLT (×10^9^/L)	0.837	0.783–0.881	<0.001	476	65.91	90.48
POD14 D-D (mg/L)	0.767	0.708–0.820	<0.001	15.99	77.27	72.38
PVD (mm)	0.742	0.682–0.797	<0.001	14	78.03	62.86
PVV (cm/s)	0.686	0.623–0.745	0.001	23.2	74.24	57.14
PVF (ml/min)	0.671	0.607–0.731	0.002	888	53.03	87.62
SVD (mm)	0.742	0.682–0.797	<0.001	10	58.33	75.24
Logit *P*	0.952	0.916–0.975	<0.001	−0.32	90.91	86.67

*PVST, portal vein system thrombosis; POD, postoperative day; AUC, area under curve; CI, confidence interval; Logit P, combined prediction of PVST based on meaningful factor.*

**Table 6:** Combined prediction of PVST based on meaningful factors called Logit *P.*^a^

The value of cut-off	Cases	PVST(n)	Non-PVST(n)	Sensitivity (%)	Specificity (%)	Positive prediction value (%)	Negative prediction value (%)	Accuracy (%)	AUROC
≥−0.32	135	120	15	–	–	–	–	–	0.916–0.975
<−0.32	102	12	90	–	–	–	–	–	–
Total	237	132	105	90.91	86.67	88.89	88.24	88.61	0.952

^*a*^ *Only one overall value for sensitivity, specificity, positive predictive value, negative predictive value, and accuracy due to calculations by the corresponding equations according to the correct number within cut-off values.*

### Single-Factor Linear Regression Analysis Performed in the Plasma of PLT and D-D

**Figure 6:**
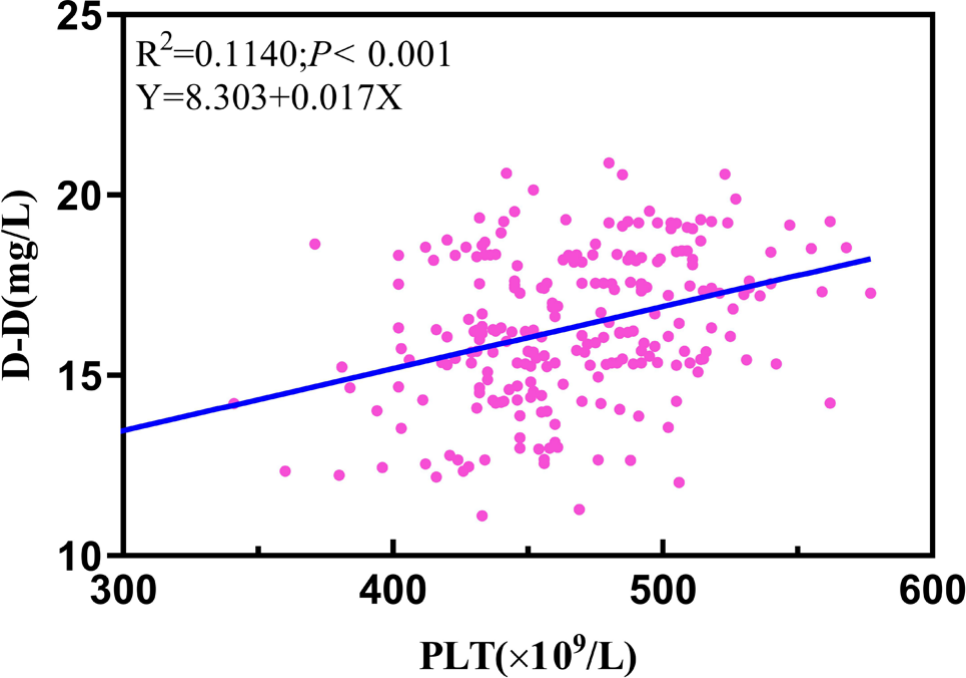
Linear regression analysis performed in the plasma of PLT and D-D. 14th D-D: postoperative 14th day of D-dimer; 7th PLT: postoperative 7th day of platelet count.

In our research, a meaningful regression equation was established (*F *= 30.242, *P *< 0.001). It demonstrated a statistical difference between platelets and D-D (*β *= 8.303; *t *= 5.449; *P *< 0.001), implying an increase in the plasma of PLT for every unit (1 × 10^9^/L) along with a parallel increase in D-D by 0.017 units (Figure 6).

## Discussion

Cirrhosis is common in most kinds of chronic liver diseases. The long course of WD with cirrhosis is characterized by complications associated with liver insufficiency, diffuse liver injury, and particularly portal hypertension (13, 14). Portal hypertension is frequently ascribed to presinus obstruction caused by pseudolobule formation in disease, resulting in an increased hepatic vascular resistance index and portal venous blood flow. Its pathologic basis is identified as decreased sensitivity to intrahepatic vasodilators such as NO, prostacyclin-2, and endothelial-dependent hypertrophy factors (15). However, there are three dangers of portal hypertension: (1) establishing collateral circulation of the portal body extremely increases the risk of gastrointestinal bleeding because of this sickness, particularly in patients with esophagogastric varices. Hypovolemic shock could happen and may lead to death without an appropriate treatment in time. Kawanaka et al. demonstrated that the incidence of gastrointestinal bleeding was as high as 25% (16). (2) Liver injury was exacerbated by establishing “splenic artery blood stealing syndrome (SASS)”. This phenomenon can be explained as follows. On the one hand, patients have a significant shortage of blood flow in the hepatic artery as a consequence of increased liver resistance index caused by portal hypertension during the cirrhosis process. On the other hand, splenic artery thickening and the resulting relatively low resistance are more likely to occur due to hypersplenism and hyperemia. Based on the two cases above, hepatic artery is disadvantageous in terms of competing with splenic artery for blood flow from the abdominal trunk, contributing to hepatocyte damage and abnormal liver function because of liver tissue ischemia and hypoxia (17, 18). (3) Hypersplenism could result in the presence of reduced whole blood cells, particularly PLT and WBC. The course of Wilson’s diseased copper could not be completed in the remaining steps. However, splenectomy has the advantage of not only decreasing portal hypertension and improving portal hemodynamics but also allowing for continued progression of WD (19).

Our results indicated that portal venous blood velocity, flow, and inner diameter decreased significantly from the preoperative 1st to the postoperative 14th day with a maximum on the day before surgery and minimum on the 14th day after surgery, and a pairwise comparison revealed a significant difference between each time point (*P *< 0.05). This result implies that surgery could cut off the blood flowing back through the splenic vein to reduce portal vein blood flow and relieve portal hypertension for gastrointestinal bleeding and hypersplenism. In addition, it could increase the portal vein blood flow into the liver for patients undergoing pericardial devascularization, as decreased portal vein collateral circulation reduces the rate of hepatic encephalopathy to some extent (20, 21). Earlier studies have revealed that splenectomy combined with pericardial devascularization proved to effectively improve liver function and portal hemodynamics with enhanced blood coagulation function among patients with liver cirrhosis and portal hypertension (22). Splenectomy for hypersplenism and gastroesophageal varices secondary to Wilson’s cirrhosis and portal hypertension is one of the mainstream treatments at present, but portal PVST formation has become the most common and dangerous problem. Clinical experience concluded that the invasion range of PVST obstruction mostly affects the splenic vein, mesenteric vein, and main portal vein, including the left and right branches, hence impairing the prognosis of patients. In our work, the incidence of PVST following splenectomy in WD patients was 55.7%, and the incidence of the portal vein, combined with splenic vein thrombosis, was 20.68% among them. The main explanation for this is that PVST patients have relatively hidden symptoms, and thrombosis is often underestimated due to immature detection methods. Therefore, abdominal ultrasound detection and CT surveillance should be routinely performed for suspected thrombosis after surgical procedures, which may greatly improve the possibility of discovering PVST formation. The clinical manifestations of PVST are linked to the obstruction of location and degree. The incident that occurs in the main portal vein not only reduces liver blood flow, resulting in liver function damage and liver failure, but also increases the risk of liver transplantation. Notably, death caused by jaundice and ascites may occur following splenectomy if there is no timely detection of PVST formation. Patients with a thrombus in the mesenteric vein, in particular, may experience intestinal obstruction and even necrosis. Simultaneously, if the splenic vein is obstructed, the clinical symptoms are atypical, manifesting as fever with a body temperature of over 38 °C (23). Here, we propose the concept “heparin antipyretic” due to the satisfactory therapeutic impact of heparin while excluding infection.

At present, different risk factors of thrombosis have been identified following splenectomy in patients with liver cirrhosis with portal hypertension, including spleen size, emodynamics, injury of the portal vein intima, changes in the blood coagulation mechanism (platelets and coagulation factors), and the mode of operation (24–26). According to our multivariate analysis, the levels of PLT and D-D on the 7th and 14th days after the operation, as well as the preoperative diameter of the splenic vein and portal vein diameter, velocity, and flow, were the influencing factors of PVST formation after surgery. In addition, ROC curves revealed that the plasma levels of PLT > 476 × 10^9^ /L on the 7th day after surgery and D-D > 15.99 mg/L on the 14th day after surgery, portal vein diameter >14 mm, velocity <23.2 cm/s, flow <888 mL/min, and splenic vein diameter >10 mm are all positively related to PVST formation.

PVST formation is mainly restricted by three factors: (1) hemodynamic alterations. Increased portal venous pressure in cirrhosis was linked to an increased risk of PVST incidents based on the theory of markedly slowing down portal vein blood flow, obstructing liver blood return, aggravating hepatic congestion, and causing compensatory portal vein widening (27). The possible reasons are as follows: first, due to structural changes in the liver, the resistance of blood flow increases to a great extent since portal vein blood flow decreases following splenectomy as the fundamental point with portal vein widening as a means of relieving pressure, causing the level of plasma tangible components in the blood, is more liable to agglutinate and deposit (28). Second, the larger the diameter of the splenic vein is, the more easily the blood flow in the vein will maintain stasis, and the more serious the wound surface of the damaged vascular inner wall will be. Because intraoperative ligated splenic vein stump forms a “blind bag” and causes “eddy current” after operation, PVST is easier to existed under this condition in the postoperative hypercoagulable environment (29). Previous studies emphasized that slow blood flow and widened veins in portal hypertension led to slow clearance of clotting substances in the blood, consistent with our result that influencing factors for PVST formation are preoperative splenic vein diameter, portal vein diameter, velocity, and flow, with OR values of 1.671, 1.565, 0.578, and 0.987, respectively (30, 31). Therefore, for clinicians, patients with the above factors should be vigilant and evaluated in time during the perioperative period if this phenomenon happens. (2) The change in the mechanism of the blood coagulation system also restricted PVST formation. PLT and D-Dimer levels after surgery are critical in reflecting coagulation function. The substance D-D, which is a specific degradation product of fibrin monomer cross-linked by activating factor XIII and hydrolyzed by plasmin, is a particular marker of fibrinolysis because its increase can directly reflect secondary fibrinolytic activity enhancement (32). A significant difference in D-D levels was observed from other reports between PVST and non-PVST groups on the 5th day after the operation in patients with cirrhotic portal hypertension (33). Similarly, our work demonstrated that the difference in D-D levels was statistically significant on the 7th and 14th days after operation. In particular, the level of D-D reached a maximum value on the 14th day postoperatively with an OR value of 1.846, which is vital in predicting thrombosis formation and could be used as a predictor of PVST after splenectomy. Currently, there is controversy regarding the relationship between PLT increase and PVST occurrence, and even opposite conclusions are reached, since there may be a relationship between the function of platelets and the degree of activation (34). Wei et al. reported that P-selectin (P-sel) participated in thrombosis initiation through mediating vascular endothelial cell adhesion and linking leukocytes to platelets (35). Nevertheless, there was a large difference in PLT levels on the 3rd and 7th days after operation. In particular, PLT revealed the highest level on the 7th day postoperatively with an OR value of 1.069. Given that filter holes on the wall of the splenic sinus can filter out aging platelets and red blood cells under normal circumstances, the level of platelets rises rapidly in the absence of a spleen. The coagulation system is activated, particularly for larger-volume PLT enzymatic active reactions, containing more dense particles, α-particles, and high-activity proteases compared with small ones, releasing more thrombus precursor substances such as thromboxane after surgery (36–39). As a result, we must be highly alerted to patients whose platelet count has risen sharply after surgery and take preventive measures in time. Furthermore, as demonstrated in our work, the primary outcome revealed that PLT level is positively correlated with D-D level on the postoperative 7th day. We indicate a rise in PLT plasma for every unit (1 × 10^9^ L) along with a parallel increase in D-D substance by 0.017 units. However, P-sel is highly expressed before surgery as a specific indicator of platelet activation function (3). Injuries of the inner wall of the vein also help PVST formation. The smooth muscles lining the vein wall are motivated to proliferate, thicken, and cause damage by portal hypertension, which also contributes to thrombus formation according to atherosclerotic alterations, collagen fiber exposure, and blood cell adhesion (40, 41). However, this factor was not identified as a risk factor in the univariate analysis, which may be linked to our sample size selection.

In long-term clinical practice, we have found that PVST formation can be effectively prevented by careful preparation in the perioperative period, while the above risk factors occur. The routine method of Doppler ultrasound examination is performed before the operation. Following this, the appropriate intervention should be actively taken aiming at 2–3 high-risk factors of portal vein diameter >14 mm, flow velocity <23.2 cm/s, flow <888 ml/min, and splenic vein diameter >10 mm. Intraoperative maneuvers should be gentle, avoiding excessive traction of the splenic vein, while also reducing intima damage. When the splenic vein is ligated during surgery, it should be as close as possible for inferior mesenteric vein and bifurcation of vein to shorten the remaining vein of spleen and alleviate hemodynamic changes. According to our clinical experience, it is highly suggested that 4000 iu of low-molecular-weight heparin should be injected subcutaneously for q12 h in the early stage combined with urokinase 200,000 units when the platelet count exceeds 476 × 10^9^/L or the partial thromboplastin time exceeds two times the normal value after surgery.

## Conclusion

In summary, splenectomy for WD can reduce portal vein velocity, flow, and inner diameter to relieve portal hypertension. Our work revealed that the PVST group had the highest level of PLT on the 7th day and D-D on the 14th day after surgery, which constituted the influencing factor of PVST formation combined with portal vein velocity, flow, inner diameter, and splenic vein inner diameter. The constructed ROC curve indicated the cut-off points of PLT at 476 × 10^9^/L, D-D at 15.99 mg/L, portal vein diameter at 14 mm, velocity at 23.2 cm/s, flow at 888 mL/min, and splenic vein diameter at 10 mm, which can be used as a clinical reference. Logit P more sensitively reflects PVST incidence based on the logistic regression prediction model with a cut-off value of −0.32 and an AUROC and an accuracy of 0.952% and 88.61%, respectively. However, it is crucial to construct an individual nomogram model to facilitate discrimination and calibration, whereas clinical validity with larger sample sizes is ensured to confirm these findings. Simultaneously, animal experiments are required to confirm the molecular mechanism underlying thrombosis.

## Data Availability

The original contributions presented in the study are included in the article/Supplementary Material; further inquiries can be directed to the corresponding author/s.
