# Supplementary material for: Research on Portal Venous Hemodynamics and Influencing Factors of Portal Vein System Thrombosis for Wilson’s Disease after Splenectomy
Source: Front Surg. 2022 May 30;9:834466. doi: 10.3389/fsurg.2022.834466 (PMC9189385; doi:10.3389/fsurg.2022.834466)
Supplement: Supplementary file 1 [file Data_Sheet_1_v1.pdf]

安徽中医药大学第一附属医院伦理审批表

主要研究者姓名/职称	于庆生(主任医师)		
项目名称	探讨肝豆状核变性脾切除术后门静脉血流动力学并分析门静脉系统血栓形成影响因素		
资助来源	国家自然科学基金 81403406		
批准文号	2019AH-32		
批准日期	2010 年 12 月 31 日		
方案版本号	V1.0	方案版本日期	2010.1.15
知情同意书版本号	V1.0	知情同意书版本日期	2010.1.15
本院承担机构及科室	安徽中医药大学第一附属医院普外一科		
研究开始和结束时间	2010 年 1 月至 2019 年 12 月		

方案设计类型：（在适当项目内打勾）

✧ ☐实验研究

✧ ☑观察性研究：☐回顾性分析　☑前瞻性研究，☐横断面研究

✧ ☐利用人体组织和信息的研究：☐以往采集保存，☐研究采集

送审材料信息

1.临床研究方案（版本号 V1.0；版本日期 2010 年 01 月 15 日）

2.知情同意书（版本号 V1.0；版本日期：2010 年 01 月 15 日）

3.病例报告表

4.伦理审批表

临床研究与方案

✧ 1.主题：探讨肝豆状核变性脾切除术后门静脉血流动力学并分析门静脉系统血栓形成影响因素

✧ 方法：所有入组患者在脾切除术后均行彩色多普勒超声监测 PVST。比较两组门静脉血液动力学指标的结果。采用受试者工作特征(ROC)曲线分析，分析各危险因素的截止值。通过单因素和多因素分析探讨 PVST 的危险因素，建立多因素诊断模型。

✧ 纳入标准和排除标准

✧ 纳入标准：

✧ 确诊 Wilson 患者（血清铜蓝 1.6μmol/24h、肝铜>250μg/g 且存在 K-F 环）

✧ 经肝脏多普勒超声、CT 或 MRI 声证实存在肝硬化及门静脉高压。

✧ 存在中重度脾功能亢进，且 WBC＜3×109/L,PLT＜60×109/L。

✧ 骨髓穿刺提示：骨髓增生象。

- ✧ 术前肝功能在 Child-Pugh 评分 8 分以内，凝血功能正常且病例资料完善。
- ✧ .排除标准：
- ✧ 既往行脾动脉栓塞术或经颈静脉肝内门体分流术(TIP)。
- ✧ 合并严重的血液系统疾病及免疫系统疾病如特发性血小板性紫癜、霍奇金淋巴瘤等。
- ✧ HBV、酒精性、血吸虫及自身免疫肝炎导致的肝硬化。
- ✧ 术前门静脉系统已有血栓及椎体外系症状严重不能行手术者。

数据的收集

- ✧ 收集所有符合标准的 Wilson 病患者的临床资料:通过临床数据库收集患者的一般资料特征、手术指标、肝功能、血常规，并在术前、术后 1、7、14 天采用彩色多普勒超声动态监测门静脉血流动力学指标。

研究申明和利益冲突

- ✧ 该项目需要记录基本临床信息，监测人体肝功能、血常规和血液动力学指标。该项目不会增加受试者的医疗费用和疼痛。研究材料和结果用于科学研究目的，不存在利益冲突。

总结

- ✧ 在本研究中，受试者的权益得到了充分的保护，对受试者没有潜在风险。大家同意，该项目的现场工作应按计划进行。

研究信息

- ✧ 资金来源：☐企业，☐政府，☐学术团体，☑本单位，☐自筹
- ✧ 数据与安全监察委员会：☑有，☐无
- ✧ 其他伦理委员会对该项目的否定性、或提前中止的决定：☑无,☐有（提交相关文件）
- ✧ 研究需要使用人体生物标本：☐否，☑是→填写下列选项：
- ✧ 采集生物标本：☑是，☐否
- ✧ 利用以往保存的生物标本：☐是，☑否

知情同意的过程

- ✧ 谁获取知情同意:☑医生/研究者,☐医生,☐研究者,☐研究护士,☐研究助理
- ✧ 获取知情同意地点:☐私密房间/受试者接待室,☐诊室,☑病房
- ✧ 知情同意签字:☑受试者签字,☐法定代理人签字

知情同意的例外：☑否,口是→填写下列选项

✧ ☐申请开展在紧急情况下无法获得知情同意的研究：

➢ 研究人群处于危及生命的紧急状况,需要在发病后很快进行干预；

➢ 在该紧急情况下,大部分病人无法给予知情同意,且没有时间找到法定代理人；

➢ 缺乏已被证实有效的治疗方法,而试验药物或干预有望挽救生命,恢复健康或减轻病痛

✧ ☐申请免除知情同意·利用以往临床诊疗中获得的病历生物标本的研究；

✧ ☐申请免除知情同意·研究病历/生物标本的二次利用；

✧ ☐申请免除知情同意签字·签了字的知情同意书会对受试者的隐私构成不正当的威胁,联系受试者真实身份和研究的唯一记录是知情同意文件,并且主要风险就来自于受试者身份或个人隐私的泄露；

✧ ☐申请免除知情同意签字·研究对受试者的风险不大于最小风险,并且如果脱离“研究”背景,相同情况下的行为或程序不要求签署书面知情同意。如访谈研究/电话调查，研究仅仅涉及摘录受试者病案的数据。

道德委员会声明

✧ 请遵循 GCP 原则和伦理委员会商定的方案开展临床研究，保护受试者的健康和权利。

✧ 要求申请人尽可能在研究开始前完成临床试验注册。

✧ 如果在研究过程中主要研究人员发生变更，以及临床研究方案、知情同意书、招募材料等方面发生变更，请申请人完善修改审查申请。

✧ 这项研究那些不符合入选标准和排除标准,满足终止试验,但没有让受试者退出研究，或给出错误的治疗或剂量，或协议禁止的药物组合，没有按照协议进行研究，或者可能对研究对象的权益和研究的科学性造成不利影响等。请积极汇报的向伦理委员会汇报情况。

✧ 如果您想暂停或终止本临床研究，请及时完善终止报告。如果您想完成临床研究，请提交结案报告。

主要研究者信息

✧ 利益冲突声明：☑ 本人与该研究项目不存在利益冲突，

☐本人与该研究项目存在利益冲突

✧ 主要研究者负责的在研项目数：3 项

✧ 主要研究者负责的在研项目中，与本项目的目标疾病相同的项目数：3 项

主要研究者声明

我保证以上信息真实准确，并负责该临床试验全过程中的质量保证，承诺该临床试验数据真实可靠，操作规范，符合 SFDA《药物临床试验质量管理规范》(GCP)要求。如有失实，愿意承担相关责任。

主要研究者签名：[signature] 联系电话： 日期：

项目研究人员列表

姓名	职称	GCP 培训（年）	研究岗位
郑州	主治医师	1	中医药在普通外科疾病中的应用

于庆生	主任医师	2	中医药在普通外科疾病中的应用
彭辉	主治医师	1	中医药在普通外科疾病中的应用
张万宗	主治医师	1	中医药在普通外科疾病中的应用
沈毅	副主任医师	1	中医药在普通外科疾病中的应用
冯辉	主治医师	1	中医药在普通外科疾病中的应用
黄龙	主治医师	2	中医药在普通外科疾病中的应用
周富海	主治医师	1	中医药在普通外科疾病中的应用
张琦	副主任医师	1	中医药在普通外科疾病中的应用
王琴	副主任医师	1	中医药在普通外科疾病中的应用

伦理委员会全体委员名单以及到会委员签名表

姓名	性别	专业情况	职务	签名
侯　勇	男	中医外科	主任委员	[illegible]
陶　永	男	中医内科心血管方向	副主任委员	[illegible]
彭长林	男	针灸	副主任委员	[illegible]
鲍　风	男	神经外科	委员	请假
张永先	男	植物保护	委员	张永先
周其艳	女	中西医结合	委员	请假
韩宁林	女	中医内科急症	委员	[illegible]
李雪苓	女	中医内科	委员	李雪苓
江树连	男	中医骨伤	委员	请假
彭学斌	男	法律	委员	请假
武　菁	女	历史	委员	武菁
张皖东	男	中医内科风湿病方向	委员	张皖东
韩燕全	男	中药师	委员	请假
张　娟	女	中医内科脑病方向	委员	[illegible]

伦理委员会盖章	科室科盖章
安徽中医药大学第一附属医院 [illegible] 委员会<br>签字：[illegible]<br>安徽省中医院）医学	安徽中医药大学第一附属医院 [illegible]<br>签字：[illegible]
