# Supplementary material for: Research on Portal Venous Hemodynamics and Influencing Factors of Portal Vein System Thrombosis for Wilson’s Disease after Splenectomy
Source: Front Surg. 2022 May 30;9:834466. doi: 10.3389/fsurg.2022.834466 (PMC9189385; doi:10.3389/fsurg.2022.834466)
Supplement: Supplementary file 4 [file Data_Sheet_4_v1.docx]

**The name of project:Research on portal venous blood flow dynamics and influencing factors of** **portal vein system thrombosis for Wilson's disease after splenectmy**

**Case Report Form**

Category:PVST□ NON-PVST□

**The name abbreviation of patient:**

**Research institutions:**

**The researchers:**

**The First Affiliated Hospital of Anhui University of Chinese Medicine**

The number/ date of version:**January 15, 2010/V1.0**

The instructions for filling in the Case Report form

1.Please use a black ballpoint or carbon pen to fill in forcefully.

2.Content Filled in must be accurate and clear without obliterating, If a mistake is happened, the researcher should underline it in the center associate with noting the correction and signing the name and time of the correction. Don’t cover up the original data, disable eraser, or draw many lines.giving an example：32.6 37.5^ZHG 2003.04.12。^

3.Every page of the CRF must be completed, and all items must be filled in.

Fill in “×” in “□” to indicate this option, such as:“×”or appropriate characters in the corresponding blanks afterwards.(1) If this item is "not done,"It is expressed by entering a character of "ND"; "Don't know" ,entering "UK"; (2) "Unable to provide" or "Not applicable''" entering "NA."The dates on all forms are expressed in the form of "month/day/year", including the patient's date of birth.If you don’t know the specific date, please use "UK" to indicate,In conclusion, please fill in the complete date as much as possible.

4.Please fill in the sign of “ND” if all inspection items are not checked or missed due to some reasons and”NK” if the specific dosage and time are unknown.Spaces such as are reserved in the CRF where values need to be filled.Please fill in the one's digit in the rightmost space, if there is a space on the left, please fill in "0".

5.The adverse event record form should be filled in truthfully during the clinical study. If there is a major unfriendly event (needing to be hospitalized, prolonged hospitalization time, disability, impacting on work ability, life-threatening or death, leading to congenital malformations),  it, in addition to addition to filling in the serious hostile event record form, must be reported to the principal investigator and the ethics committee and related departments in time.

**Clinical Study Flow chart**

| [stage](D:/LenovoSoftstore/Install/wangyiweidaocidian/8.9.9.0/resultui/html/index.html#/javascript:;) | Screening program | therapy | | follow-up visit | | |
| --- | --- | --- | --- | --- | --- | --- |
| [doctors'](D:/LenovoSoftstore/Install/wangyiweidaocidian/8.9.9.0/resultui/html/index.html#/javascript:;) [office](D:/LenovoSoftstore/Install/wangyiweidaocidian/8.9.9.0/resultui/html/index.html#/javascript:;) [visiting](D:/LenovoSoftstore/Install/wangyiweidaocidian/8.9.9.0/resultui/html/index.html#/javascript:;) | First diagnose | [perioperative](D:/LenovoSoftstore/Install/wangyiweidaocidian/8.9.9.0/resultui/html/index.html#/javascript:;) [period](D:/LenovoSoftstore/Install/wangyiweidaocidian/8.9.9.0/resultui/html/index.html#/javascript:;) | | [the](D:/LenovoSoftstore/Install/wangyiweidaocidian/8.9.9.0/resultui/html/index.html#/javascript:;) [first](D:/LenovoSoftstore/Install/wangyiweidaocidian/8.9.9.0/resultui/html/index.html#/javascript:;) [time](D:/LenovoSoftstore/Install/wangyiweidaocidian/8.9.9.0/resultui/html/index.html#/javascript:;) | [The second time](D:/LenovoSoftstore/Install/wangyiweidaocidian/8.9.9.0/resultui/html/index.html#/javascript:;) | [The](D:/LenovoSoftstore/Install/wangyiweidaocidian/8.9.9.0/resultui/html/index.html#/javascript:;) [third](D:/LenovoSoftstore/Install/wangyiweidaocidian/8.9.9.0/resultui/html/index.html#/javascript:;) [time](D:/LenovoSoftstore/Install/wangyiweidaocidian/8.9.9.0/resultui/html/index.html#/javascript:;) |
| The time of visiting | 3～0 day | [pre-operation](D:/LenovoSoftstore/Install/wangyiweidaocidian/8.9.9.0/resultui/html/index.html#/javascript:;) | [pos- operation](D:/LenovoSoftstore/Install/wangyiweidaocidian/8.9.9.0/resultui/html/index.html#/javascript:;) | The first day after surgery±2day | The 1th week±2day | The 2th week±2天 |
|  | | | | | | |
| Signing of the informed consent | × | × |  |  |  |  |
| Determining inclusion/exclusion | × | × |  |  |  |  |
| Splenectomy | × | × | |  |  |  |
| Physical indicators | | | | | | |
| The routine of blood | × | × | × | × | × | × |
| The routine of coagulation | × | × | × | × | × | × |
| Liver function（ALT,AST,etc） | × | × | × | × | × | × |
| renal function（BUN,Cr,etc） | × | × | × | × | × | × |
| The electrolyte(Na,K,etc) | × | × | × | × | × | × |
| Blood glucose and lipid | × | × | × | × | × | × |
| The routine of urine | × | × | × | × | × | × |
| The examination of stool | × | × | × | × | × | × |
| Electronic gastroscopy and pathological examination | × |  |  |  |  | × |
| Digestive tract angiography |  |  |  |  |  |  |
| The abdominal computed tomography (CT) | × |  |  |  |  |  |
| Electrocardiogram (ecg) | × | × |  |  |  |  |
| Anteroposterior radiograph of the chest | × | × |  |  |  |  |
| 评价指标 | | | | | | |
| The time of operation(min) |  | × | |  |  |  |
| The blood loss of operation |  | × | |  |  |  |
| Adverse event assessment | × | × | × | × | × | × |
| The reason analysis of shedding |  |  |  | × |  |  |
| Comprehensive curative effect evaluation |  | | × | × | × | × |

The records of observation during the enrollment period

Date of diagnosis:Month /day /year

The basic information and clinical data of subjects

| Name： | | The contact phone number |  | | | |
| --- | --- | --- | --- | --- | --- | --- |
| The source of the patient | □Outpatient treatment □hospitalization（number： ） | | | | | |
| The address of home |  | | | | | |
| gender | □Male □female | | | | | |
| The date of birth | Month /day /year | | Age | | Year | |
| Body height | \|_\|_\|_\|.\|_\| cm | | Body weight | | \|_\|_\|_\|.\|_\| kg | |
| Body temperature | \|_\|_\|_\| ℃ | | breathing | | \|_\|_\| Times/min | |
| The pulse rate | \|_\|_\|_\| Times/min | | Blood pressure | | \|_\|_\|_\|/\|_\|_\|_\|mm Hg | |
| nation |  | | BMI（kg/m2） | | \|_\|_\|_\| | |
| [underlying](D:/LenovoSoftstore/Install/wangyiweidaocidian/8.9.9.0/resultui/html/index.html#/javascript:;) [disease](D:/LenovoSoftstore/Install/wangyiweidaocidian/8.9.9.0/resultui/html/index.html#/javascript:;) | hypertension□ diabetes□ ascites□ other□ | | | | | |
| The classification of The Child | A□ | | | B□ | | C□ |
| Surgical method | Pericardiac vascularization+ splenectomy□ | | | Splenectomy□ | | Other□ |
| Operative skills | Traditional splenectomy□ | | | Anatomic splenectomy□ | | Other□ |
| The time of operation (min) |  | | | | | |
| Intraoperative blood loss(ml) |  | | | | | |

Diagnosis and course of disease

| Western medicine diagnosis: Wilson's disease □  TCM syndrome differentiation: accumulation □  The course of the disease： month |
| --- |

Treatment history of this disease:Yes □ No□,If yes, please complete the following form:

| The ways of treatment | [therapeutic](D:/LenovoSoftstore/Install/wangyiweidaocidian/8.9.9.0/resultui/html/index.html#/javascript:;) [schedule](D:/LenovoSoftstore/Install/wangyiweidaocidian/8.9.9.0/resultui/html/index.html#/javascript:;) | Start date(Month /day /year) | [ending](D:/LenovoSoftstore/Install/wangyiweidaocidian/8.9.9.0/resultui/html/index.html#/javascript:;) [date](D:/LenovoSoftstore/Install/wangyiweidaocidian/8.9.9.0/resultui/html/index.html#/javascript:;) |
| --- | --- | --- | --- |
|  |  |  |  |
|  |  |  |  |
|  |  |  |  |
|  |  |  |  |
|  |  |  |  |

The patients have other diseases at the time of enrollment:Yes □ No□,If yes, please complete the following form:

| [sequence](D:/LenovoSoftstore/Install/wangyiweidaocidian/8.9.9.0/resultui/html/index.html#/javascript:;) [number](D:/LenovoSoftstore/Install/wangyiweidaocidian/8.9.9.0/resultui/html/index.html#/javascript:;) | [name](D:/LenovoSoftstore/Install/wangyiweidaocidian/8.9.9.0/resultui/html/index.html#/javascript:;) [of](D:/LenovoSoftstore/Install/wangyiweidaocidian/8.9.9.0/resultui/html/index.html#/javascript:;) [disease](D:/LenovoSoftstore/Install/wangyiweidaocidian/8.9.9.0/resultui/html/index.html#/javascript:;) |
| --- | --- |
| 1 |  |
| 2 |  |
| 3 |  |

The patient have any combination of other drugs at the time of enrollment:Yes □ No□,If yes, please complete the following form:

| TThe name of drug | [rReasons](D:/LenovoSoftstore/Install/wangyiweidaocidian/8.9.9.0/resultui/html/index.html#/javascript:;) [for](D:/LenovoSoftstore/Install/wangyiweidaocidian/8.9.9.0/resultui/html/index.html#/javascript:;) [using](D:/LenovoSoftstore/Install/wangyiweidaocidian/8.9.9.0/resultui/html/index.html#/javascript:;) | TThe dosage | Start date of medication (Month /day /year) |
| --- | --- | --- | --- |
|  |  |  |  |
|  |  |  |  |
|  |  |  |  |

| **[Research](D:/LenovoSoftstore/Install/wangyiweidaocidian/8.9.9.0/resultui/html/index.html#/javascript:;)** **[project](D:/LenovoSoftstore/Install/wangyiweidaocidian/8.9.9.0/resultui/html/index.html#/javascript:;)** | | | | | | | |
| --- | --- | --- | --- | --- | --- | --- | --- |
| TThe  [bblood](D:/LenovoSoftstore/Install/wangyiweidaocidian/8.9.9.0/resultui/html/index.html#/javascript:;) [examination](D:/LenovoSoftstore/Install/wangyiweidaocidian/8.9.9.0/resultui/html/index.html#/javascript:;) |  | Before | POD1 | POD3 | POD7 | POD14 | POD21 |
|  | ALB(g/L) |  |  |  |  |  |  |
|  | AST(U/L) |  |  |  |  |  |  |
|  | ALT(U/L) |  |  |  |  |  |  |
|  | PLT(×10^9^/L) |  |  |  |  |  |  |
|  | WBC(×10^9^/L) |  |  |  |  |  |  |
|  | HB(g/L) |  |  |  |  |  |  |
|  | FIB(g/L) |  |  |  |  |  |  |
|  | TBIL(μmmol/L) |  |  |  |  |  |  |
|  | APTT(s) |  |  |  |  |  |  |
|  | PT(s) |  |  |  |  |  |  |
|  | D-D(mg/L) |  |  |  |  |  |  |
| [imageological](D:/LenovoSoftstore/Install/wangyiweidaocidian/8.9.9.0/resultui/html/index.html#/javascript:;) [examination](D:/LenovoSoftstore/Install/wangyiweidaocidian/8.9.9.0/resultui/html/index.html#/javascript:;) | Portal vein diameter(mm) |  |  |  |  |  | ND |
|  | Portal vein **[velocity](D:/LenovoSoftstore/Install/wangyiweidaocidian/8.9.9.0/resultui/html/index.html#/javascript:;)** (cm/s) |  |  |  |  |  | ND |
|  | [portal vein flow](D:/LenovoSoftstore/Install/wangyiweidaocidian/8.9.6.0/resultui/html/index.html#/javascript:;)(ml/min) |  |  |  |  |  | ND |
|  | Splenic vein diameter(mm) |  |  |  |  |  | ND |
| Complications (number of cases) | [anastomotic](D:/LenovoSoftstore/Install/wangyiweidaocidian/8.9.9.0/resultui/html/index.html#/javascript:;) [fistula](D:/LenovoSoftstore/Install/wangyiweidaocidian/8.9.9.0/resultui/html/index.html#/javascript:;) | NO□ | | | Yes□ | | |
|  | [Intra-abdominal Hemorrhage](D:/LenovoSoftstore/Install/wangyiweidaocidian/8.9.9.0/resultui/html/index.html#/javascript:;) | NO□ | | | Yes□ | | |
|  | [abdominal infection](D:/LenovoSoftstore/Install/wangyiweidaocidian/8.9.9.0/resultui/html/index.html#/javascript:;) | NO□ | | | Yes□ | | |
|  | [pulmonary](D:/LenovoSoftstore/Install/wangyiweidaocidian/8.9.9.0/resultui/html/index.html#/javascript:;) [infection](D:/LenovoSoftstore/Install/wangyiweidaocidian/8.9.9.0/resultui/html/index.html#/javascript:;) | NO□ | | | Yes□ | | |
|  | [infection](D:/LenovoSoftstore/Install/wangyiweidaocidian/8.9.9.0/resultui/html/index.html#/javascript:;) [of](D:/LenovoSoftstore/Install/wangyiweidaocidian/8.9.9.0/resultui/html/index.html#/javascript:;) [incisional](D:/LenovoSoftstore/Install/wangyiweidaocidian/8.9.9.0/resultui/html/index.html#/javascript:;) [wound](D:/LenovoSoftstore/Install/wangyiweidaocidian/8.9.9.0/resultui/html/index.html#/javascript:;) | NO□ | | | Yes□ | | |
|  | [urinary](D:/LenovoSoftstore/Install/wangyiweidaocidian/8.9.9.0/resultui/html/index.html#/javascript:;) [system](D:/LenovoSoftstore/Install/wangyiweidaocidian/8.9.9.0/resultui/html/index.html#/javascript:;) [infection](D:/LenovoSoftstore/Install/wangyiweidaocidian/8.9.9.0/resultui/html/index.html#/javascript:;) | NO□ | | | Yes□ | | |
|  | [portal vein system thrombosis](D:/LenovoSoftstore/Install/wangyiweidaocidian/8.9.9.0/resultui/html/index.html#/javascript:;) | NO□ | | | Yes□[location](D:/LenovoSoftstore/Install/wangyiweidaocidian/8.9.9.0/resultui/html/index.html#/javascript:;)： | | |
|  | death | NO□ | | | Yes□ | | |

Note：;BeforeThe :preoperative first day;POD:postoperative day.

Subject inclusion judgment

| Inclusion criteria | | Yes | No |
| --- | --- | --- | --- |
| (1)Patients diagnosed with Wilson's disease(Serum copper blue 1.6μmol/24h, liver copper >250μg/g and K-F ring).  (2)The presence of cirrhotic portal hypertension was confirmed by hepatic Doppler ultrasound, CT or MRI.  (3)Moderate and severe hypersplenism within White Blood Cell(WBC)＜3×109/L,Platelets(PLT)＜60×109/L.  (4) Bone marrow hyperplasia is suggested by Bone marrow puncture.  (5)The score of preoperative liver function was less than 8 points in Child-Pugh with normal coagulation function and complete data of case. | | □  □  □  □  □  □ | □  □  □  □  □  □ |
| [Exclusion](D:/LenovoSoftstore/Install/wangyiweidaocidian/8.9.9.0/resultui/html/index.html#/javascript:;) [criteria](D:/LenovoSoftstore/Install/wangyiweidaocidian/8.9.9.0/resultui/html/index.html#/javascript:;) | | Yes | No |
| (1)Splenic artery embolization or transjugular intrahepatic portosystemic shunt (TIP) was performed before.  (2)Combined with serious blood system diseases and immune system diseases such as idiopathic thrombotic purpura and Hodgkin's lymphoma.  (3)Liver cirrhosis caused by HBV, alcoholic, schistosomiasis and autoimmune hepatitis.  (4)Patients with preoperative portal vein thrombosis and severe extraspinal symptoms which that cannot be operated on. | | □  □  □  □  □  □ | □  □  □  □  □  □ |
| Determinine whether the subjects are included | □ Yes □ No | | |

The place where the test sheet is pasted

| Date of final visit | (Month /day /year ) |
| --- | --- |
| Date of last visit | (Month /day /year ) |
| The completion of the study | □completion □suspending（Please choose the reasons for the suspension) |
| Reasons for discontinuing the study | □[adverse](D:/LenovoSoftstore/Install/wangyiweidaocidian/8.9.9.0/resultui/html/index.html#/javascript:;) [event](D:/LenovoSoftstore/Install/wangyiweidaocidian/8.9.9.0/resultui/html/index.html#/javascript:;) □protocol deviation  □[loss](D:/LenovoSoftstore/Install/wangyiweidaocidian/8.9.9.0/resultui/html/index.html#/javascript:;) [to](D:/LenovoSoftstore/Install/wangyiweidaocidian/8.9.9.0/resultui/html/index.html#/javascript:;) [follow-up](D:/LenovoSoftstore/Install/wangyiweidaocidian/8.9.9.0/resultui/html/index.html#/javascript:;)□Withdrawal of informed consent in a midway study  □The study as a whole was suspended □The Lack of efficacy  □Others →elaborating: |
| Suspending the patient firstly | □Patients (including their guardians) □Experimental investigator □The sponsor  □□Others →elaborating： |
| Adverse events during the study | □No □Yes→filling in the "Adverse Event Form" |
| Drug combination during the study | □No □Yes→filling in the"Combined medication list" |

The summary table of the end of the study

Effectiveness evaluation

It shall be completed uniformly by statisticians according to standards.

The signature of  doctor Month /day /year

The record form of combined treatment methods

| The way of treatment |  | The start time of treatment |  | The end time of treatment |
| --- | --- | --- | --- | --- |
|  |  |  |  |  |
|  |  |  |  |  |
|  |  |  |  |  |
|  |  |  |  |  |
|  |  |  |  |  |
|  |  |  |  |  |
|  |  |  |  |  |
|  |  |  |  |  |

NOTE：No therapeutic drugs and treatment methods with similar functions as the test should be used in combination during the study.

Combined with other diseases and treatment records

| Complicating with other diseases | The way of treatment | The time of onset of illness |
| --- | --- | --- |
|  |  |  |
|  |  |  |
|  |  |  |
|  |  |  |
|  |  |  |
|  |  |  |
|  |  |  |
|  |  |  |

Adverse event report form

| （Standard medical terms）Record all observed results by asking the sentences such as "How do you feel differently since your last check?".Using diagnostic names rather than symptom names whenever possible.One adverse event was recorded in each column. | | | |
| --- | --- | --- | --- |
| The Clinical symptoms of AE |  |  |  |
| The medical System of AE |  |  |  |
| The time of AE | Month /day /year | Month /day /year | Month /day /year |
| The vanishing time of AE | Month /day /year | Month /day /year | Month /day /year |
| The characteristics of AE | Paroxymoron→attack：□□/time  □persistent | Paroxymoron→attack：□□/time  □persistent | Paroxymoron→attack：□□/time  □persistent |
| The extent of the AE | □Mild  □Moderate  □Severe | □Mild  □Moderate  □Severe | □Mild  □Moderate  □Severe |
| The ending event caused by AE | □hospitalization  □Extending hospital stay  □Disability  □Dysfunction  □Congenital malformations  □Life-threatening  □Death  □No | □hospitalization  □Extending hospital stay  □Disability  □Dysfunction  □Congenital malformations  □Life-threatening  □Death  □No | □hospitalization  □Extending hospital stay  □Disability  □Dysfunction  □Congenital malformations  □Life-threatening  □Death  □No |
| The prognosis of AE | □Disappear → sequela:  Yes□No□  □Continue  □Death | □Disappear → sequela:  Yes□No□  □Continue  □Death | □Disappear → sequela:  Yes□No□  □Continue  □Death |
| The measures taken by surgical methods | □Continue the operation  □Stop the operation  □Other conservative treatment options | □Continue the operation  □Stop the operation  □Other conservative treatment options | □Continue the operation  □Stop the operation  □Other conservative treatment options |
| The relationship with surgical methods | □sure  □probable  □[unrelati](D:/LenovoSoftstore/Install/wangyiweidaocidian/8.9.9.0/resultui/html/index.html#/javascript:;)on  □[Immeasurement](D:/LenovoSoftstore/Install/wangyiweidaocidian/8.9.9.0/resultui/html/index.html#/javascript:;) | □sure  □probable  □[unrelati](D:/LenovoSoftstore/Install/wangyiweidaocidian/8.9.9.0/resultui/html/index.html#/javascript:;)on  □[Immeasurement](D:/LenovoSoftstore/Install/wangyiweidaocidian/8.9.9.0/resultui/html/index.html#/javascript:;) | □sure  □probable  □[unrelati](D:/LenovoSoftstore/Install/wangyiweidaocidian/8.9.9.0/resultui/html/index.html#/javascript:;)on  □[Immeasurement](D:/LenovoSoftstore/Install/wangyiweidaocidian/8.9.9.0/resultui/html/index.html#/javascript:;) |
| Treatment | □Yes □No | □Yes □No | □Yes □No |
| Quit the test | □Yes □No | □Yes □No | □Yes □No |

The signature of  doctor Month /day /year
