# Supplementary material for: Research on Portal Venous Hemodynamics and Influencing Factors of Portal Vein System Thrombosis for Wilson’s Disease after Splenectomy
Source: Front Surg. 2022 May 30;9:834466. doi: 10.3389/fsurg.2022.834466 (PMC9189385; doi:10.3389/fsurg.2022.834466)
Supplement: Supplementary file 5 [file Data_Sheet_5_v1.docx]

**Anhui Provincial Traditional Chinese Hospital Institutional Review Board Approval Form（IRB）**

Our hospital plans to carry out the scientific research work of " Research on portal venous blood flow dynamics and influencing factors of portal vein system thrombosis for Wilson's disease after splenectmy:a retrospective cohort study". The ethics committee of our hospital has reviewed the medical ethics issues related to the project.

**Project information:**

Project: Research on portal venous blood flow dynamics and influencing factors of portal vein system thrombosis for Wilson's disease after splenectmy.

The approval number: 2019AH-32

The approval date: December 31, 2010

Project Leader：Zhou Zheng

Institution: The first hospital affiliated to Anhui University of Chinese Medicine

Professional ranks and titles：Attending doctor

**Clinical investigation and schem:**

Objective: Research on portal venous blood flow dynamics and influencing factors of portal vein system thrombosis for Wilson's disease after splenectmy

Methods: Color Doppler ultrasonography was performed for monitoring PVST in all enrolled patients after splenectomy. Outcomes of hemorheological indexes were compared between groups. Receiver operating characteristic (ROC) curve analysis was conducted to analyze the cutoff value of each risk factor. Univariate and multivariate analyses were carried out to explore risk factors of PVST.

Inclusion and exclusion criteria: [Inclusion](D:/LenovoSoftstore/Install/wangyiweidaocidian/8.9.5.0/resultui/html/index.html#/javascript:;) [criteria](D:/LenovoSoftstore/Install/wangyiweidaocidian/8.9.5.0/resultui/html/index.html#/javascript:;):(1)Patients diagnosed with Wilson's disease(Serum copper blue 1.6μmol/24h, liver copper >250μg/g and K-F ring).(2)The presence of cirrhotic portal hypertension was confirmed by hepatic Doppler ultrasound, CT or MRI.(3)Moderate and severe hypersplenism within White Blood Cell(WBC)＜3×109/L,Platelets(PLT)＜60×109/L.(4) Bone marrow hyperplasia is suggested by Bone marrow puncture.(5)The score of preoperative liver function was less than 8 points in Child-Pugh with normal coagulation function and complete data of case.[Exclusion](D:/LenovoSoftstore/Install/wangyiweidaocidian/8.9.5.0/resultui/html/index.html#/javascript:;) [criteria](D:/LenovoSoftstore/Install/wangyiweidaocidian/8.9.5.0/resultui/html/index.html#/javascript:;):(1)Splenic artery embolization or transjugular intrahepatic portosystemic shunt (TIP) was performed before.(2)Combined with serious blood system diseases and immune system diseases such as idiopathic thrombotic purpura and Hodgkin's lymphoma.(3)Liver cirrhosis caused by HBV, alcoholic, schistosomiasis and autoimmune hepatitis.(4)Patients with preoperative portal vein thrombosis and severe extraspinal symptoms which that cannot be operated on.

Data collection during routine procedures.Clinical data were collected from all patients:[character](D:/LenovoSoftstore/Install/wangyiweidaocidian/8.9.9.0/resultui/html/index.html#/javascript:;) of general information, surgical indexes,liver function,blood routine was collected by Clinical database and the hemodynamic indexes of the portal vein were dynamically monitored by color Doppler ultrasound on the preoperative, postoperative 1,7 and14 day

Starting and finishing time of the study：January 2010 to December 2019

**The main contents of human body research are as follows:**

The project needs to record basic clinical information and monitor the human liver function,blood routine and hemorheology indicators. The project does not increase the medical expenses and pain of the subjects. The research materials and results are used for scientific research purposes, and there is no conflict of interest.

**Comments on ethical review:**

After deliberation by the ethics committee of our hospital, the experimental design and scheme of the study fully considered the principles of safety and fairness, and the research content would not cause harm and risk to the subjects. The recruitment of subjects will be based on the principle of voluntary and informed consent, and the rights and privacy of the subjects will be protected to the maximum extent. There will be no conflict of interest between the research content and the research results.

**Conclusion：**

In this study, the rights and interests of the subjects were fully protected and there was no potential risk to the subjects. It was agreed that the on-site work of the project should be carried out as planned.

**Statement of ethics committee:**

Please follow the GCP principle and the protocol agreed by the ethics committee to carry out clinical research, and protect the health and rights of the subjects.

Applicants are requested to complete the clinical trial registration as much as possible before the start of the study.

If the main investigator is changed during the study, and any changes are made to the clinical study protocol, informed consent, recruitment materials, etc., the applicant is requested to improve the application for amendment review.

The study included subjects who did not meet the inclusion criteria or the exclusion criteria, met the termination of the trial but did not let the subjects withdraw from the study, given wrong treatment or dosage, given the combination drugs prohibited by the protocol, etc., and did not follow the protocol to carry out the study, or may cause adverse effects on the rights and interests of the subjects and the scientific nature of the study. Please apply for the situation that violates the GCP principle.

If you want to suspend or terminate the clinical study, please improve the termination report in time. If you want to complete the clinical study, please submit the closing report.

Term of validity: January 2010 to December 2019

Signature of principal investigator:

Director of ethics committee:

Ethics Committee of Anhui Hospital of Traditional Chinese Medicine
