# Supplementary material for: Research on Portal Venous Hemodynamics and Influencing Factors of Portal Vein System Thrombosis for Wilson’s Disease after Splenectomy
Source: Front Surg. 2022 May 30;9:834466. doi: 10.3389/fsurg.2022.834466 (PMC9189385; doi:10.3389/fsurg.2022.834466)
Supplement: Supplementary file 6 [file Data_Sheet_6_v1.doc]

**The Signature of Informed Consent**

**The subject statement**

I have had the opportunity to ask questions and all questions have been answered after having read this informed consent carefully. Since this participation in this experiment is voluntary,I can choose not to participate or withdraw at any time during this research without discrimination or retaliation upon notification to the investigator.however,Any of my medical benefits and benefits will not be affected.If I require additional diagnosis/treatment, either I do not comply with the study plan or for other reasonable reasons, the investigator may discontinue my continued participation in the study.If I need to take any other medications due to a change in my condition, I will consult my doctor in advance afterwards.

I have consented to the review of my research materials by The departments of drug supervisory and administrative and the Ethics Committee

I consent to □ or reject □ the use of my medical records for research other study than present

The signature of the subject (or the legal agents) date

Subjects contact phone number:

**The investigator Statement**

I have accurately informed the subjects of the informed consent and answered their questions.The result expessed that the subjects volunteered to participate in this clinical trial.

The signature of the doctor date

The doctor work phone number:
